# SUSTAINABILITY OF COMPREHENSIVE DEMENTIA CARE INTERVENTIONS FROM A CLINICAL PERSPECTIVE

**DOI:** 10.1093/geroni/igae098.0257

**Published:** 2024-12-31

**Authors:** Gary Epstein-Lubow, Ellen Tambor, Maddie Johnson, Kristin Lees Haggerty, Rebecca Stoeckle

**Affiliations:** Education Development Center, Waltham, Massachusetts, United States; Education Development Center, Waltham, Massachusetts, United States; Education Development Center, Waltham, Massachusetts, United States; Education Development Center, Waltham, Massachusetts, United States; Education Development Center, Waltham, Massachusetts, United States

## Abstract

Multiple evidence-based comprehensive dementia care programs are currently available for adoption by healthcare systems and providers. However, barriers to adoption and long-term sustainability remain. The need for effective payment policies to incentivize implementation of comprehensive dementia care has long been recognized and the recently announced Center for Medicare and Medicaid (CMS) Guiding an Improved Dementia Care Experience (GUIDE) Model is an important step forward in improving access to comprehensive dementia care for Medicare beneficiaries and family caregivers. The GUIDE Model seeks to confirm that comprehensive dementia care can be widely implemented without raising health care costs. Similar work is needed under Medicare Advantage and other health plans. The National Dementia Care Collaborative (NDCC) seeks to accelerate dissemination of six effective comprehensive dementia care programs. The NDCC provides a forum for addressing barriers to adopting and sustaining comprehensive dementia care and seeks to promote that GUIDE Model participants use at least one evidence-based program. Existing evidence-based dementia care models, developed and tested within large health systems and academic medical centers, are now demonstrating ability to adapt to the characteristics of diverse service-delivery organizations leading to widespread adoption and sustainability. To succeed, one high priority is expanding the dementia care workforce to deliver high quality, comprehensive dementia care. Also essential is consensus on a core set of metrics for monitoring the quality and fidelity of comprehensive dementia care.

